# Stakeholder diversity matters: employing the wisdom of crowds for data-poor fisheries assessments

**DOI:** 10.1038/s41598-024-84970-4

**Published:** 2025-01-02

**Authors:** Benjamin L. H. Jones, Rolando O. Santos, W. Ryan James, Samuel Shephard, Aaron J. Adams, Ross E. Boucek, Lucy Coals, Sophia V. Costa, Leanne C. Cullen-Unsworth, Jennifer S. Rehage

**Affiliations:** 1 Project Seagrass, Bridgend, UK; 2 Department of Earth and Environment, Institute of Environment, Florida International University, Miami, FL USA; 3 Seagrass Ecosystem Research Group, Department of Biosciences, Swansea University, Swansea, UK; 4 Department of Biological Sciences, Institute of Environment, Florida International University, Miami, FL USA; 5 Inland Fisheries Ireland, Dublin, Ireland; 6 Ave Maria University, Ave Maria, FL USA; 7 Bonefish and Tarpon Trust, Miami, FL USA; 8 Florida Atlantic University Harbor Branch Oceanographic Institute, Fort Pierce, FL USA; 9 School of Life and Environmental Sciences, Deakin University, Geelong, VIC Australia

**Keywords:** Collective intelligence, Fisheries management, Indigenous and local knowledge, Recreational fisheries, Wisdom of crowds, Ecology, Environmental social sciences

## Abstract

Embracing local knowledge is vital to conserve and manage biodiversity, yet frameworks to do so are lacking. We need to understand which, and how many knowledge holders are needed to ensure that management recommendations arising from local knowledge are not skewed towards the most vocal individuals. Here, we apply a Wisdom of Crowds framework to a data-poor recreational catch-and-release fishery, where individuals interact with natural resources in different ways. We aimed to test whether estimates of fishing quality from diverse groups (multiple ages and years of experience), were better than estimates provided by homogenous groups and whether thresholds exist for the number of individuals needed to capture estimates. We found that diversity matters; by using random subsampling combined with saturation principles, we determine that targeting 31% of the survey sample size captured 75% of unique responses. Estimates from small diverse subsets of this size outperformed most estimates from homogenous groups; sufficiently diverse small crowds are just as effective as large crowds in estimating ecological state. We advocate for more diverse knowledge holders in local knowledge research and application.

## Introduction

A framework for using the UN Decade of Ocean Science for Sustainable Development in support of policy and action highlights the importance of integrating different forms of knowledge to conserve and manage biodiversity^1^. Indigenous and local knowledge (ILK) is one such form of knowledge, held by communities of people who live or work in, interact with, and/or make their livelihoods from natural resources and includes the cumulative body of localised and site-specific knowledge about the relationship of living beings with one another and with their environment^2^. In fisheries science, the contributions of ILK towards filling data gaps and providing new knowledge are numerous^3–5^, yet examples of incorporation into fisheries management are few^6^. Despite multiple calls to bridge, or interweave ILK with standard scientific knowledge for management purposes numerous challenges have prevented this from happening^7–11^.

Appropriate frameworks are lacking for how to integrate ILK within management^7,12,13^, but it is important to (a) ascertain that local knowledge is of interest, and (b) interweave this knowledge with standard scientific knowledge. To meet these two aims, it is vital to understand which^14^, and how many knowledge holders are appropriate to derive robust and sufficient understanding of a local system^15^. For devising management strategies, this is inherently important because targeting the wrong individuals may strongly influence findings and a skewed sample may completely change outcomes of a study. Across the breadth of ILK and expert knowledge research however, transparency and structured frameworks in study design are lacking^16,17^. A review of fisheries-focused ILK research found that knowledge holders were diverse, and that methods as well as sample sizes varied considerably among studies^6^. This review showed that in the last 10 years, nearly 80% of interview studies gave no justification for sample size, and instead took a sampling approach of interviewing as many as or all experts possible. As argued by Martin, et al.^18^, further research is required to determine who should be consulted, the number of individuals needed, and which methods should be used for combining expert judgments to ascertain ecological state. Such research could be theoretical or applied but would greatly enhance our ability to interweave ILK with standard scientific knowledge or to monitor and manage social-ecological systems based solely on ILK.

Individuals use multiple mental tools to form and process knowledge, solve problems, and make decisions (e.g., logic, statistics, perspectives, heuristics)^19^. Over the last few decades, interdisciplinary research has emerged revealing the value of collective approaches to solve complex problems, for example, generating quantifiable estimates or making predictions about the future^20–22^. Woolley, et al.^23^, define a group’s collective intelligence (CI) as the ‘general capacity of a group to perform a wide variety of tasks.’ Such work posits that leveraging the diverse problem-solving abilities of a collective, the CI of individuals surpasses the problem-solving capabilities of a single individual or expert. These benefits are a result of *superadditivity*, that is, the notion that the combinations of the mental tools that we use can be more powerful than the individual tools themselves^24^. We, like others^25^, argue that collective approaches are untapped and extend to ILK.

Wisdom of Crowds (WoC) is one form of CI suggesting that the amalgamation of a diverse crowd’s predictions may yield considerably more accurate results than those produced by single experts (an individual or a homogenous group). This takes an asynchronous approach, whereby individually obtained information is aggregated with no social interactions^26^. The first use of this principle was by Galton^27^ in 1906, where estimates of the weight of an ox at a county fair from 787 fairgoers were analysed. Unsurprisingly, no individual estimated correctly, but when estimates were averaged, the *vox populi* (i.e., average from independent estimates), was within 0.8% of the true value^27^. Arlinghaus and Krause^28^ argue that this approach should be applied to fisheries, for example, by asking individuals to generate estimates of absolute stock sizes or biomass.

While one might argue that a WoC approach is not dissimilar to other forms of knowledge elicitation, the principles defined by Surowiecki^26^ provide a clear framework that sets it apart from how ILK is currently elicited. These principles are stakeholder diversity, stakeholder independence, decentralization, aggregation, and trust. Firstly, diversity of opinion is the most important element of a WoC approach. Elicitation methods must ensure that the collective is a diverse mix of individuals who encounter and interact with natural resources in various ways (e.g., for livelihood or for recreation). Research on this topic reveals that groups of highly diverse individuals can outperform similar-sized but more homogeneous groups of scientifically trained individuals^29^. This is in contrast to traditional forms of ILK elicitation, particularly those involving Indigenous peoples, where the underlying process is typically to target a homogenous group comprised of the oldest or most experienced individuals, those often considered to be most knowledgeable on an ecological subject^14^. Second, Surowiecki^26^ argues that there must be independence of opinion within groups, that is, there should be limited social interactions among participants during the process. Minimising the effect of opinion leaders is crucial to ensure that diversity of opinion is sustained^30^, and methodically, individuals should be elicited through diverse and multiple networks or approaches. The third principle outlined is decentralization, that is, individuals should be motivated and encouraged to specialize and draw on their own knowledge (e.g., how do *you* perceive the quality of fish stocks?), rather than be presented with information that might influence their own knowledge (e.g., fish stocks are declining, do you agree or disagree?). Fourth, there must be aggregation of knowledge; a mechanism must exist for turning individual judgements into collective judgement. So, for this knowledge to be aggregated, it must be quantifiable. The fifth, and final principle is trust; each individual trusts the collective to provide honest answers.

While applications of collective approaches are still relatively new, WoC has been used in a diverse variety of fields including economics^31,32^, where social media users predicted financial trends just as accurately as professional analysts, in politics^33,34^, where individual predictions of election results were more accurate than exit polls, and in medical fields^35,36^, where crowd wisdom accurately predicted the spread and severity of COVID-19 infections before data were available. While it has been proposed as an approach for conservation management in data-poor contexts, such as fisheries^28^, it has yet to become established and few studies refer to or use a WoC framework^37,38^. In a fisheries context, a study by Gray, et al.^37^ paired local knowledge gathered through WoC with empirical data, and found that diverse recreational angler club members were able to accurately estimate the size-dependent demographics of their fishery when compared with the empirical data source. They found that aggregated estimates from just 33 individuals, when diverse enough, were able to provide accurate quantitative observations from a fishery with around 170,000 license holders. Similarly, Aminpour, et al.^38^ show that aggregating knowledge from diverse resource users through graphical mental models produced a social–ecological system model comparable to the best scientific understanding. As argued by Arlinghaus and Krause^28^, empirical studies are needed to carefully test the potential of WoC frameworks to provide robust estimates of fishery status.

Recreational fisheries are a data-poor sector^39,40^, yet the potentially high local knowledge among the community is still relatively untapped and may well provide valuable information to contribute to fisheries management. Given the need for robust information to manage such fisheries, and to determine the who and how many in expert elicitation studies in order to obtain such robust information, we sought to apply a WoC framework to a recreational catch-and-release bonefish (*Albula vulpes*) fishery in South Florida, USA; a data-poor situation with high uncertainty and where information on the status and trends of stocks is lacking^41^. Operationalising a WoC framework and using the local knowledge held by members of this fishery, our study had three aims: (1) to analyse how estimates of fishing quality (see methods) provided by homogenous groups compared to estimates from a larger diverse group, (2) to ascertain whether there are thresholds for the number of individuals needed to capture robust estimates of fishing quality, and (3) to test the accuracy of smaller groups of varying diversity to estimate fishing quality. To meet these aims, we use a dataset of bonefish fishing quality estimates from South Florida recreational fishers^42^; estimates that have previously been quantified and match trends from other data for the region^43–47^.

## Results

### Study demographics

**Fig. 1:**
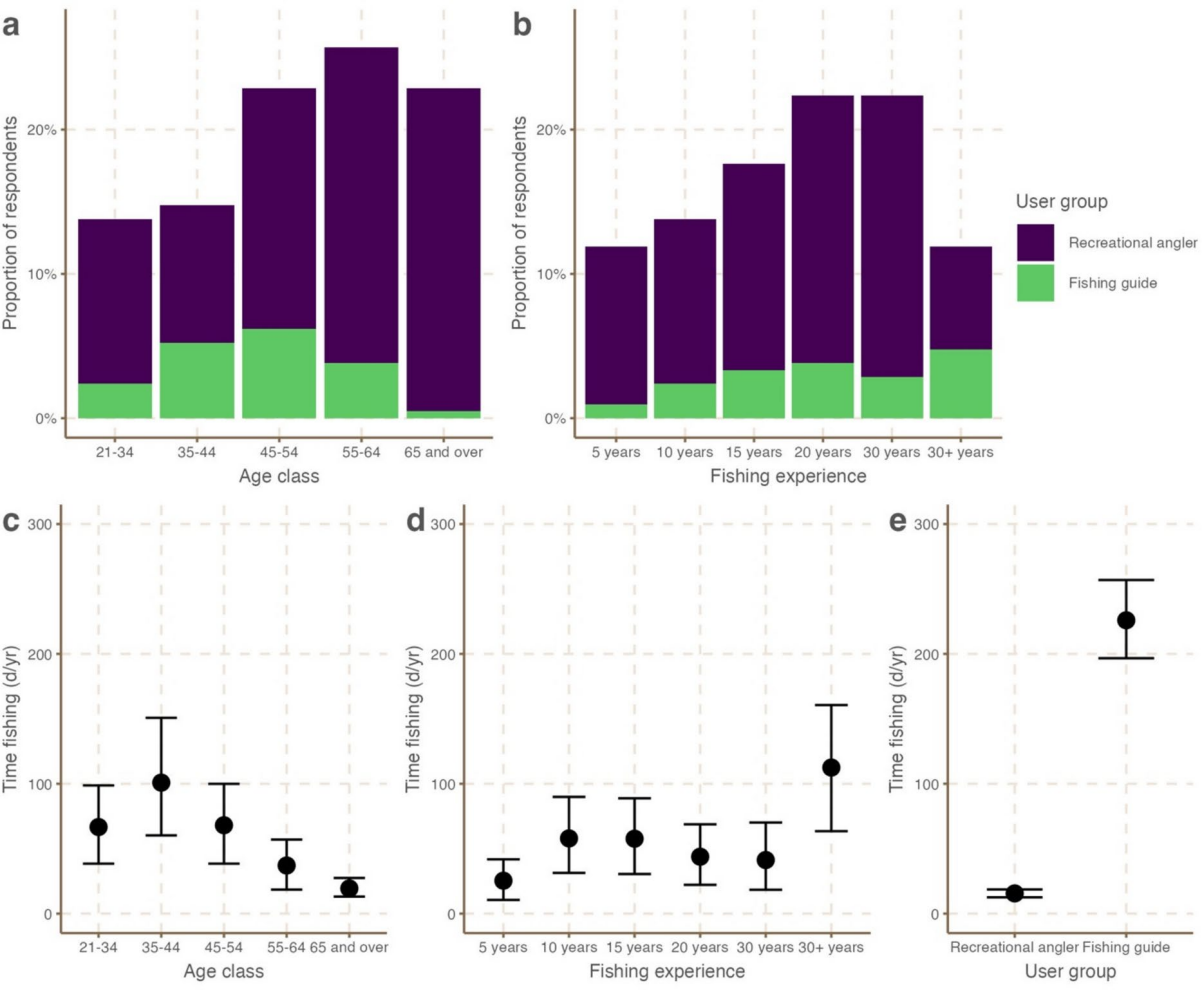
Demographics of the full crowd (n = 210 individuals). Bar plots (panels **a**, **b**) show the number of individuals across age classes and years of fishing experience. Panels **c**, **d** and **e** show the average number of days spent fishing each year across age classes, fishing experience categories and user groups. Points represent mean ± 95% confidence intervals.

For the purpose of this study, the “crowd” comprised a sample of 210 South Florida recreational anglers and fishing guides who fully completed a 2015 online survey where they were asked about the quality of bonefish fishing in South Florida from 1975 until 2015. Of our crowd, 172 individuals classified themselves as anglers, primarily using natural resources for *recreation*, and 38 classified themselves as guides, primarily using natural resources for a *livelihood*. Most respondents (71.5%) were older than 45, where respondents in the age bracket 55–65 accounted for 25.7%, followed by those in the age bracket 45–54 and 65 and over, both 22.9% (Fig. 1a). Comparatively, most fishing guides were younger than 55 and over 65% of respondents had been fishing for 20 years or less (Fig. 1b). We found no strong relationship between age and fishing experience to suggest that the two are related (Supplementary Fig. 1). Individuals from our crowd spent varying amounts of time fishing per year; younger individuals generally spent more days fishing than older individuals (Fig. 1c), while more experienced fishers spent more days fishing than less experienced fishers (Fig. 1d), and fishing guides spent 200 more days per year interacting with natural resources than recreational anglers (Fig. 1e). As defined by Surowiecki^26^, our crowd comprised a heterogeneous mix of individuals of various ages, whom have spent varying amounts of time fishing, and come into contact with natural resources in different ways.

### Wisdom of crowds on fishing quality

**Fig. 2:**
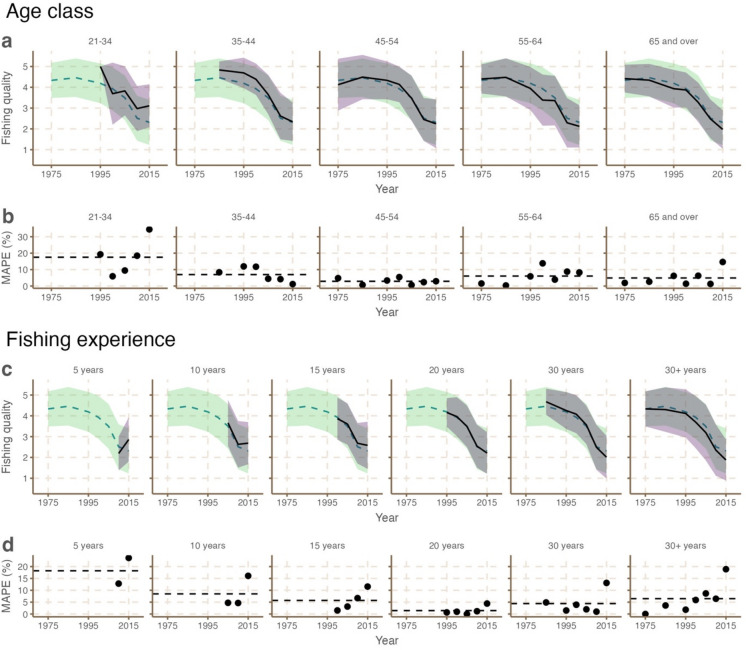
Aggregated estimates (mean ± standard deviation) of bonefish fishing quality in South Florida, USA, from 1975–2015 partitioned by age class (panel **a**) and fishing experience (panel **c**) where a value of 5 = very good and a value of 1 = very poor. In **a** and **c** the green dashed line shows the mean trend from the full sample and shaded green area shows the standard deviation from the full sample. The black line shows the mean trend from the group and shaded purple area shows the standard deviation from the group (grey where it overlaps with the results from the full sample). Panel **b** and** d** shows the mean absolute percentage error (MAPE) of estimates (compared with the full sample mean) of different groups across each time period. Black dashed lines in **b** and **d** represent the mean absolute percentage error (MAPE) of the group.

Using relative absolute error metrics (see Methods), we compared fishing quality trends produced by the full crowd (n = 210) with trends produced by homogenous groups from the same dataset (subsets based on age and experience). Different age classes of respondents detected similar trends in fishing quality (Fig. 2a). These trends were comparable to those detected by the full crowd (Supplementary Fig. 2). In contrast, mean absolute percentage error (MAPE) was different among age groups (Fig. 2b). Estimates from respondents in the age class 45–54 more closely resembled responses from the full sample but these respondents had greater within group variability in responses across time periods than any other age class. We found that respondents in the age classes 21–34 and 35–44 provided higher estimates for fishing quality in recent time points (particularly in 1995) than the full sample. Error was highest for the youngest individuals (MAPE = 17.5 ± 11.1%), but less than 10% for individuals in the age class 35–44. However, these individuals spent more time on average fishing per year than all other groups (Fig. 1c) and had a higher proportion of guides (Fig. 1a), implying that they may be better placed to accurately estimate recent fishing quality. The opposite was true for respondents in the age class 65 and over, who provided lower estimates (although not significant; < 5% error) for fishing quality in 2015 compared with the full sample; these individuals spent the least amount of time on average fishing per year but produced estimates that were similar to the full sample (< 5% error). Generally, error for each individual age class was variable, non-linear (Supplementary Fig. 3), and lowest for individuals between the ages of 45–54 (MAPE = 2.89 ± 1.86). Despite similar trends in some age groups, only those aged 45–54, and 65 and over produced estimates that were similar to the full crowd (< 5% error).

Much like age classes, parsing our analysis by fishing experience also revealed variability in aggregated estimates of fishing quality over years (Fig. 2c). Due to the nature of our methods, only respondents that had fished for 30 + years were able to provide estimates for the full period but were also the group that spent more time on average fishing per year than other groups. Despite accounting for 11.9% of respondents, within group variability was highest in this group, and estimates for recent time points were lower than those of the crowd. Fishers that had been fishing for 30 years provided higher estimates for fishing quality in 1985 than those fishing for 30 + years. Only those individuals fishing for 5 and 10 years perceived an increase in fishing quality from 2010 and 2015 and the magnitude of increase in quality was highest for those fishing for 5 and less; a group that spent the lowest amount of time fishing per year. As with age, we found that the MAPE of estimates from each individual fishing experience category were variable (Fig. 2d) and not linear across years of experience (Supplementary Fig. 3). Error was highest for the least experienced individuals (MAPE = 18.2 ± 22.7%), and lowest for individuals with 20 years of experience (MAPE = 1.44 ± 1.71%). Only groups with 20 and 30 years of experience produced estimates that were similar to the full crowd (< 5% error).

**Fig. 3:**
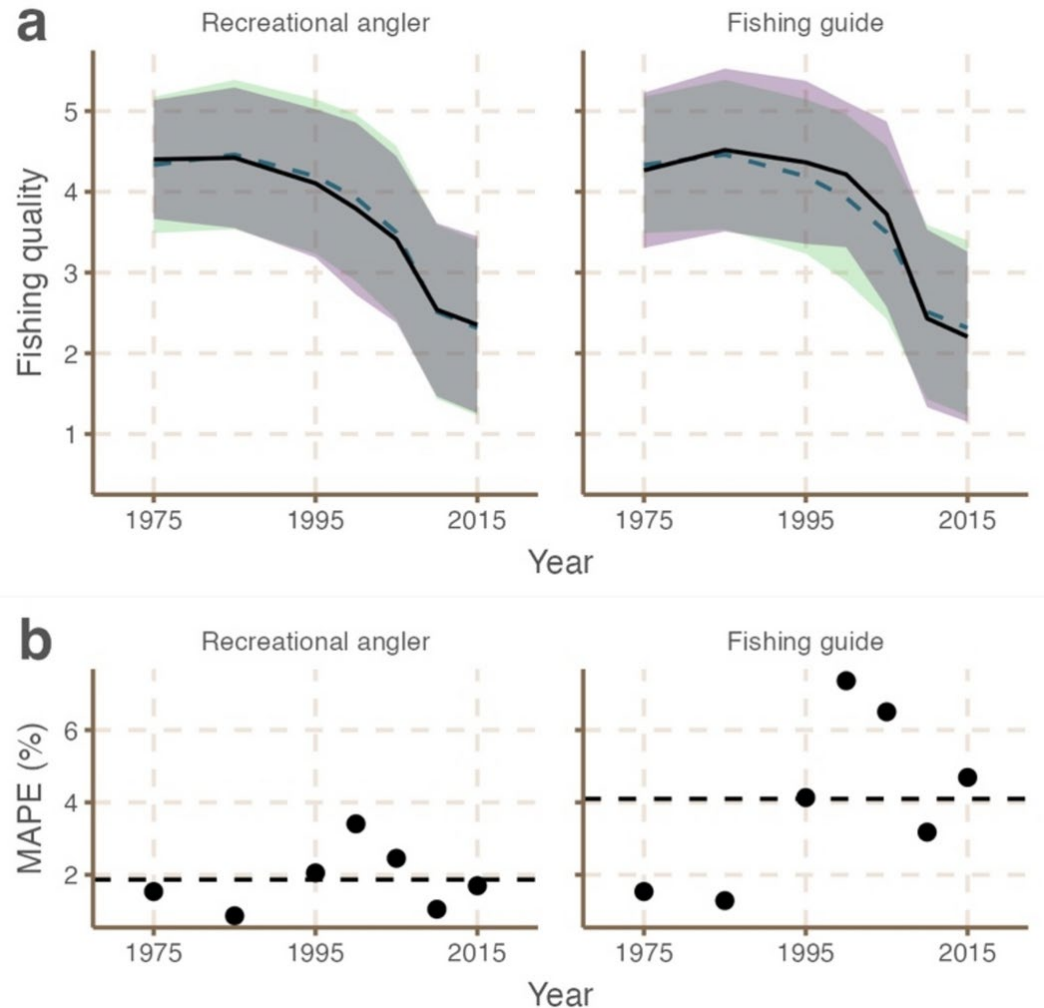
Aggregated estimates (mean ± standard deviation) of bonefish fishing quality in South Florida, USA, from 1975–2015 differentiated by recreational anglers and fishing guides (panel **a**) where a value of 5 = very good and a value of 1 = very poor. In **a** the black line shows the mean trend from the group and shaded purple area shows the standard deviation from the group. The green dashed line shows the mean trend from the full sample and shaded green area shows the standard deviation from the full sample. Panel **b** shows the mean absolute percentage error (MAPE) of estimates (compared with the full sample mean) of different groups across each time point. Black dashed lines in **b** represent mean absolute percentage error (MAPE) of the group.

Lastly, in terms of fishing quality in 1975, 2010 and 2015, the two user groups (recreational anglers and fishing guides) provided near identical estimates (Fig. 3a). However, the steepness of decline in fishing quality was more pronounced in estimates from fishing guides, although within group variability was greater for this group for older time points suggesting that there was more consensus among recreational anglers. That said, fishing guides spent over three times more days per year fishing than recreational anglers. MAPE of estimates from each user group were low (Fig. 3b). For recreational anglers, MAPE was 1.87 ± 0.87%, likely driven by high within group diversity in terms of age, fishing experience and time spent fishing (Fig. 2), but also high similarity with the full sample given that 172 individuals were anglers. Error was over twice as high for fishing guides (MAPE 4.10 ± 2.31%).

### Saturation of unique fishing quality estimates

**Fig. 4:**
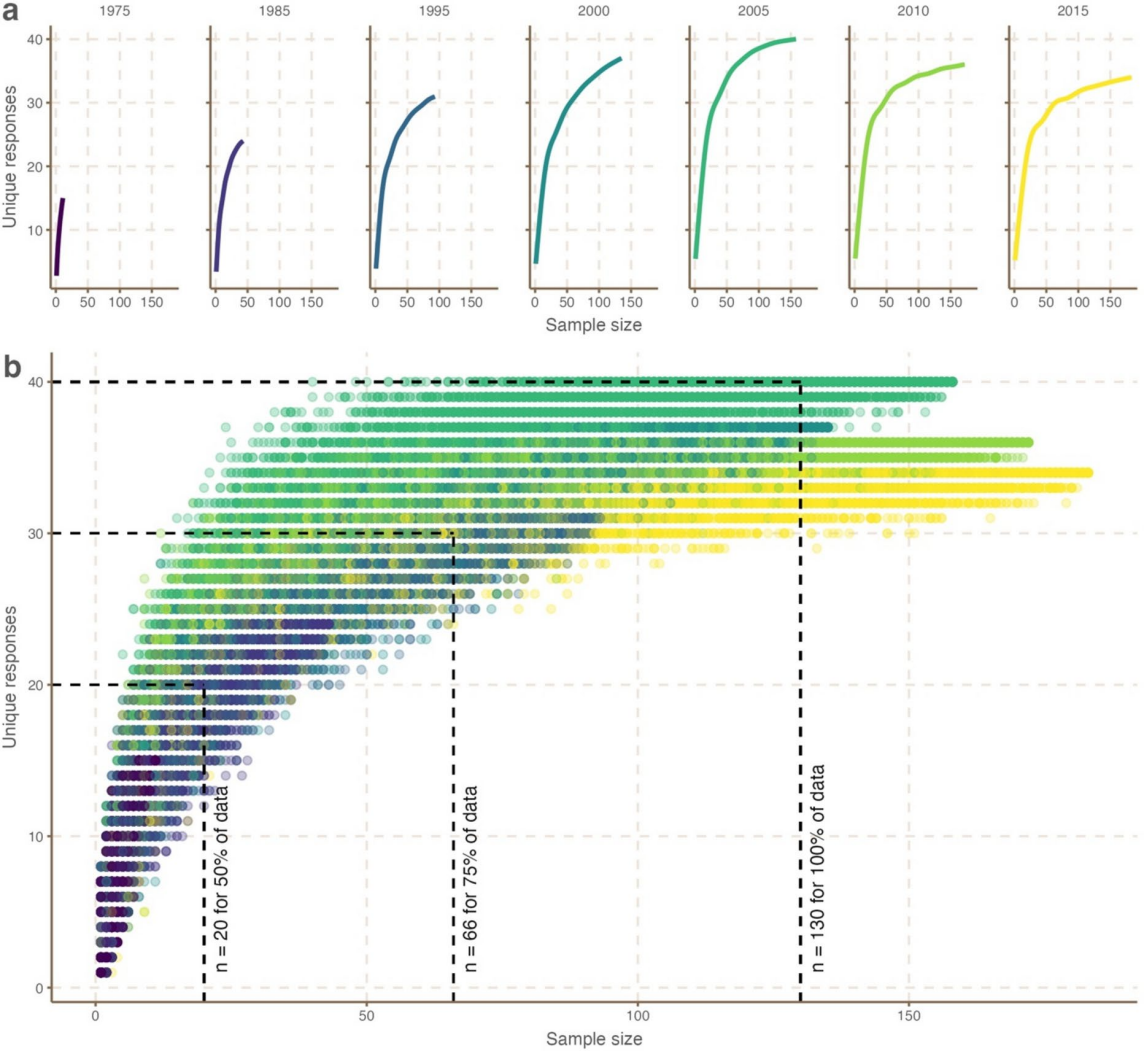
Unique responses to a question about bonefish fishing quality in South Florida between 1975 and 2015 generated by randomly selected subsets of varying sample sizes. Panel **a** shows the relationship between number of unique responses generated with increasing sample size per time point. The maximum number of unique responses generated varied across periods because information was gathered from fewer respondents in early time periods (e.g., in more distant years (1975, 1985), some respondents were not yet born or not fishing). Panel **b** shows separate saturation curves as per panel **a** for each of the seven time periods (1975–2015), and dotted lines represent the mean sample size required to capture, 50% (n = 20), 75% (n = 66) and 100% (n = 130) of unique responses. To capture all unique responses, fewer individuals were needed than our full sample (n = 210).

Given that our crowd included a heterogeneous mix of individuals of various ages who have spent varying amounts of time fishing, relatively few of our respondents were able to provide fishing quality estimates for the earliest time periods (e.g., 1975 and 1985). Based on repeated random subsampling and saturation curves, we found that only three time periods (2005, 2010, 2015) had a large enough number of active anglers and guides for the number of unique responses to level off (reach an asymptote) and reach saturation (Fig. 4). Based on the maximum number of unique responses possible, we calculated the minimum sample size needed to record 100% of unique responses to be 130 ± 21.5, far lower than our actual sample size of 210. In addition, we calculated the minimum sample size needed to record 75% of unique responses to be 66 ± 19.0, and 50% of unique responses to be 20 ± 6.03, representing 32% and 10% of our sample size respectively.

### Sample size and crowd diversity effects on bonefish fishing quality estimates

**Fig. 5:**
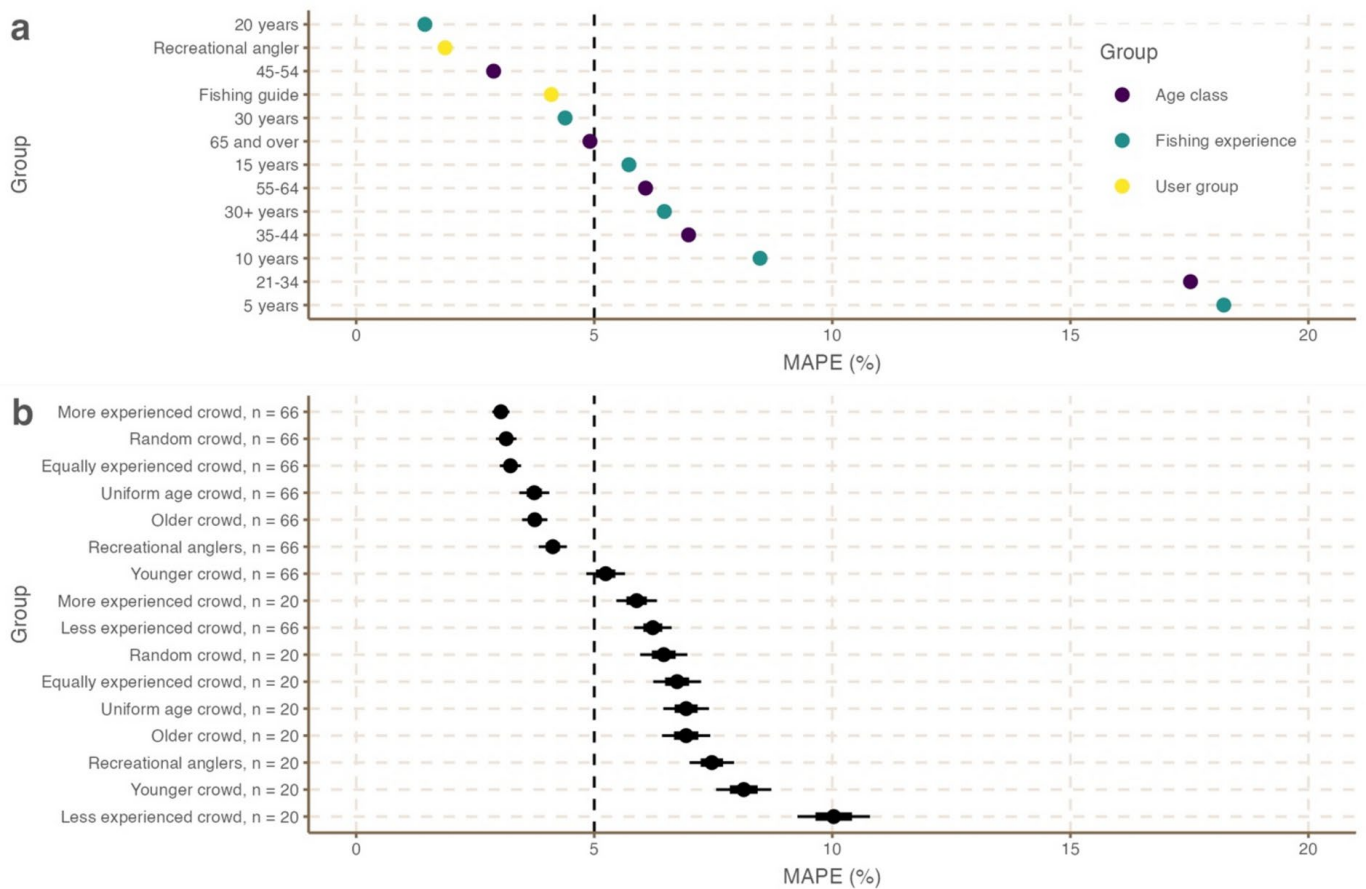
Mean absolute percentage error (MAPE) of estimates (compared with the full sample mean) of **a** homogenous groups from the full sample and **b** diverse subsets from repeated subsampling. Points in **b** are presented with standard error (thick line) and 95% confidence intervals (thin line). Dashed line represents an MAPE of 5% where points below this are considered very good estimates.

Comparing minimum sample size estimates with those of the crowd revealed that estimates from random subsets of 66 individuals (i.e., the sample size required to capture 75% of unique responses) closely resembled the decline in bonefish fishing quality over years (Supplementary Fig. 4). Random subsets of 66 individuals were also sufficient to capture responses from 1975, despite the proportionally lower number of respondents in the full sample able to provide estimates for this period. Mean absolute error estimates from 66 individuals were within 5% of the full sample (MAPE = 3.32 ± 1.12%). In contrast, estimates from random subsets of 20 individuals (i.e., the sample size required to capture 50% of unique responses) provided more variable estimates across time points, and the likelihood of gaining estimates for 1975 was reduced to 64%. Despite this, MAPE estimates from 20 individuals for each time point were still within 6.61 ± 2.24% of those provided by the full sample (Fig. 5).

Given that the variation in estimates depended on age classes and fishing experience, we then produced diverse subsets of 66 and 20 individuals, weighted either by age structure or fishing experience (see Methods). Weighting by age provided near identical trends for fishing quality when sampling 66 individuals, but only the older crowd consistently provided estimates across the full range of years (Supplementary Fig. 4). We found that the likelihood of gaining estimates for 1975 was 98% for the age-uniform crowd, and 87% for the younger crowd. The older (MAPE 3.53 ± 1.13%) and age-uniform crowd provided truer estimates (MAPE 3.55 ± 1.48%) than the younger crowd (MAPE 5.36 ± 1.88%). In contrast, when sampling 20 individuals, while the trends produced were similar to the full dataset, we found that aggregate estimates were more variable; MAPE for the age-uniform crowd increased to 7.15 ± 2.80%, increased for the older crowd to 6.59 ± 2.43% and increased for the younger crowd to 8.56 ± 3.35%. Moreover, when sampling 20 individuals, likelihood of gaining estimates for 1975 was reduced to 82% for the older crowd, 64% for the age-uniform crowd and 47% for the younger crowd. The chance of gaining estimates for 1985 also reduced in the age-uniform (99%), and younger crowd (88%).

Weighting by experience also produced near identical trends for fishing quality when sampling 66 individuals. However, as with age, only the equally experienced crowd and the more experienced crowd provided estimates across the full range of years (Supplementary Fig. 4). For the less experienced crowd, likelihood of gaining estimates from 1975 was 0%, and for 1985 was 87%. Both the equally experienced (MAPE = 3.33 ± 1.24%) and more experienced crowd (MAPE = 2.94 ± 0.85%) produced estimates that were within 5% error, whereas estimates from the less experienced crowd were higher (MAPE = 6.31 ± 1.87%). Lastly, with a sample size of 20, no single crowd was 100% likely to generate estimates for the full range of periods. Likelihood of generating responses in 1975 reduced to 97% for the older crowd, 79% for the equally experienced crowd and remained at 0% for the less experienced crowd. Further, likelihood of generating estimates for 1985 were also reduced in the equally experienced (99%) and less experienced crowd (43%), and further reduced for 1995 on the less experienced crowd (92%). We again found that aggregate estimates were more variable with a lower sample size; MAPE for the equally experienced crowd increased to 6.70 ± 2.64%, increased for the more experienced crowd to 6.17 ± 2.43% and increased for the less experienced crowd to 9.17 ± 3.16%. This suggests that if sample size is low, error can be minimized by targeting groups where older or more experienced individuals are more frequent, but if sample size is moderate, error is generally minimized with diversity.

Finally, comparing estimation error across diverse and relatively homogenous groups (Fig. 5) revealed that subsets at moderate sample sizes (e.g., those needed to capture 75% of unique data) outperformed 77% of homogenous groups and all subsets at low sample sizes. However, resource users with 20 years of fishing experience (n = 47), and users that were recreational anglers (n = 172) performed the best. The latter outcome (for recreational anglers) was likely driven by high sample size, as error was higher for subsets of 66 and 20 anglers. This was supported by the fact that subsets of recreational anglers (n = 66) performed similar to guides (n = 38). For subsets, moderately sized crowds (n = 66) of either equally experienced or more experience crowds minimized error, outperforming random, uniform-age and equally experienced crowds. Further, moderately sized subsets using experience outperformed those based on age. For smaller crowds (n = 20), performance was maximized by more experienced crowds, but at an error rate above 5%.

## Discussion

There have been numerous calls to bridge and interweave Indigenous and local knowledge (ILK) with scientific knowledge^7,10,11^. Focusing only on the local aspect of ILK, this study sought to provide insights into how the number and diversity of stakeholders influences the ability of a group to solve complex problems in social-ecological systems. In doing so, we provide guidance to scientists and practitioners using local knowledge to monitor and manage systems, but it is unclear whether these same principles are relevant to Indigenous knowledge which was not tested within this study. We applied a wisdom of crowds (WoC) framework to a recreational catch-and-release bonefish fishery with limited to no formal monitoring. Like many recreational fisheries, trends in bonefish stock status are highly uncertain, and insufficient data is available for the use of traditional stock assessment models^41^. This fishery example represents a natural resource system where users are diverse, comprised of various ages, differing levels of fishing experience and effort, and use natural resources for different reasons (recreation and livelihood). In this system, individuals interact with, observe changes in, and sample natural resources in different ways, leading to diverse perceptions about status and trends. We found that such diversity matters; aggregation of knowledge obtained from diverse groups produced status and trend information that outperformed information from more homogeneous groups.

Firstly, we found that subsets of homogenous groups of individuals (e.g., based on age class, fishing experience) produced differing estimates of fishing quality. While estimated trends were similar (all groups showed a relative decline in fishing quality), the changes observed between groups varied. In common with van den Heuvel and Rönnbäck^48^, we found that older and more experienced fishers perceived fishing quality to be worse than younger and less experienced fishers for recent timepoints. In contrast, younger fishers provided higher estimates for fishing quality than older fishers for mid time points (e.g., 1995–2000). Disparities across groups may be a consequence of differences in values and interests, differing motivations to respond to surveys that may influence management, the differing ways they use and interact with natural resources, or the ways that they communicate and share knowledge^43,49–52^. We argue that each homogenous group may hold knowledge that is biased to some degree and a myriad of different psychological mechanisms and cognitive processes exist that can either disguise or inflate trends, both at a group and individual level. On one hand, anglers and guides who have been fishing for a number of years may be influenced by ‘memory illusion’, caused by the influential memory or nostalgia of extremely good fishing days^53^, but on the other hand younger individuals might be influenced by ‘generational amnesia’ or the ‘shifting baseline syndrome’^54^.

Numerous local knowledge studies, particularly within fisheries, explicitly seek to target individuals with the most experience^14^. Such individuals are often older, and while we show that these groups are important, particularly for capturing historic information, younger individuals, who spend more time fishing are equally important. In our study, only younger and less experienced individuals perceived an increase in fishing quality from 2010 to 2015, a trend that is mirrored in tournament records from the same area^43^, but missed by older and more experienced individuals. While less experienced in terms of actual years spent fishing, younger individuals spent more time fishing per year than any other age group and are potentially much better placed to provide recent estimates.

Anglers vary in their ability to catch fish and fishing skill is influenced by numerous factors^55,56^. How skill influences success, how success influences satisfaction, and how factors other than success influence satisfaction are complex^57,58^. For reasons unknown, more experienced anglers tend to be less satisfied at similar fishing success levels than less experienced anglers^59^. Despite likely positive relationships between fishing skill, age and fishing experience^60^, these relationships are not linear. As individuals age, traits important for angling decrease (e.g., eyesight, speed, and strength). Given that personal awareness of aging is variable^61^, it is likely that fishing skill eventually decreases, despite perceived skill and years of experience being high. As such, cognitive dissonance and motivated reasoning might also influence estimates^62^, where less satisfied individuals might be motivated to reason that stocks are lower, when in fact their skill and corresponding success rate may have declined^63^.

Consistent with the ‘diversity trumps ability’ theory suggested by Page^24^, and conforming to the WoC^26^, we argue then that the aggregation of fishing quality estimates obtained from the full crowd were more accurate than those of any single homogeneous group. Aggregation of estimates from the full sample produced trends that were more reflective of the natural resource and are comparable to findings from other fisheries-dependent data for the region^42–46^. We argue then, that stakeholder diversity mediates for generational differences, ‘shifting baseline syndrome’, potential effects of ‘memory illusion’, and any other cognitive processes such as motivated reasoning.

For WoC to be successful, the process of aggregating knowledge needs to mediate for within group biases, filter out knowledge saturation within groups, and combine knowledge across multiple groups^64–66^. We investigated the optimum crowd size needed to meet these conditions by combining rarefaction methods from the ecology literature^67^ with saturation principles from the social science literature^68^. Given that rarefaction curves show how species richness changes as sampling effort increases, we use the same principle to show how response richness (e.g., unique fishing quality estimates) changes as sampling effort increases (e.g., number of individuals interviewed). Using random subsampling to produce saturation curves, we reiterated that sample size effects are important to consider when harnessing local knowledge. More importantly, we demonstrated that small groups of respondents can provide robust insights on ecological state, and that additional respondents would be unlikely to provide any new information. Specifically for our study we found that targeting 32% of the survey sample size (n = 66 respondents) captured 75% of unique fishing quality estimates across time periods. These findings mirror similar approaches that use local knowledge saturation curves to determine fishing extent^15^. A second key finding is that estimates of ecological state from small diverse subsets of respondents outperformed most estimates from homogenous groups (i.e., age class, fishing experience, user groups). Small diverse subsets were consistently similar to the large sample of individuals.

These findings reiterate the importance of diversity and that the specific characteristics of individuals targeted when conducting ILK research can strongly influence findings. This issue is particularly important for management, given that a skewed sample may completely change outcomes. This point is significant, since the ILK literature abounds with research that provides little or no information on sample determination^16,17^. Our survey relied on a multi-pronged sampling approach whereby the survey was distributed in multiple ways: (1) emails to fishing membership groups, (2) emails to fishing guide associations, (3) articles in fishing magazines, and (4) advertisements at fishing stores. This approach increased respondent diversity and reduced sampling bias^69^, possibly reducing the number of respondents needed to derive asymptotic and relatively certain estimates of fishery state.

This study has various implications for survey design, particularly in the context of ILK and WoC frameworks. Firstly, we suggest that methods to produce saturation curves can be easily applied to both quantitative and qualitative data gathered through surveys or interviews. Such analysis can be repeated throughout the course of a survey to understand whether data obtained is sufficient for stable state assessments. Second, understanding demographics of the survey population is important and should be considered prior to survey design. Fishing communities are diverse, and what that diversity looks like may not be apparent until after a study is conducted. In this example we looked at diversity in terms of age and experience, but other factors such as social networks, gear types, vessel types, distance to fishing grounds, and livelihood strategies add differing levels of stakeholder diversity to a population. If marginalised groups are present, understanding the survey population first is vital to ensure that all voices are included in a survey. This provides opportunity to (a) involve stakeholders early in the process and (b) ensure that stakeholder diversity is integrated. Thirdly, we suggest that approaches targeting smaller samples sizes can be just as powerful, but only when there is sufficient diversity in respondents. If we had used in-person interviews like most ILK research^6^, instead of an online survey, our findings suggest that we could have potentially saved resources by targeting a smaller group of individuals. Depending on elicitation method, smaller samples are also easier to compile and analyse, particularly if information needs to be coded. Lastly, we suggest that ILK approaches can increase sample diversity by broadening elicitation methods; diversified survey tools are more friendly to the diverse population.

In conclusion, we show that sufficiently diverse small crowds are just as effective as large crowds in estimating ecological state. In this case, diversity trumps ability, and we advocate for more diverse knowledge holders in ILK research, rather than solely focusing on the oldest and most experienced individuals. This example shows that WoC frameworks are powerful and suggests that similar ILK frameworks could be and should be encouraged to generate estimates of absolute stock size or biomass. Such estimates, rather than relative trend information, would allow us to interweave ILK with biomass-based reference points to manage data-poor fisheries.

## Methods

### Study system

To understand if local knowledge gathered through Wisdom of Crowds (WoC) frameworks are robust for management of social-ecological systems, we used a data-poor recreational catch-and-release bonefish (*Albula vulpes*) fishery in South Florida, USA, as a case study. The recreational bonefish fishing areas in South Florida extend approximately 400 km from Biscayne Bay to the Marquesas. Briefly, this study focused on three main regions: Biscayne Bay, Florida Bay and the Florida Keys. For full details on the study system, see Rehage, et al.^42^.

### Online survey

To quantify changes in the South Florida recreational bonefish fishery, Rehage, et al.^42^ designed an online survey relying on a targeted sampling approach to engage numerous stakeholders. Survey distribution was multi-pronged and employed (1) emails to fishing membership groups, (2) emails to fishing guide associations for distribution to their members (emails to members, websites and/or social media), (3) advertisement articles in numerous fishing magazines (print, websites and/or social media), and (4) printed advertisements at local fishing shops across South Florida (a display with business cards providing information and a link to the online survey). This approach was used to ensure that a diversity of stakeholders, and the way that they interact with information about the fishery, and each other was incorporated into soliciting responses. The online survey was open from August 2015 to January 2016.

The survey comprised 10 questions^42^, with the first section comprised of demographic questions about respondent age, gender, years of bonefishing experience and frequency, whether they guided and frequency of guiding activities. For age, respondents were asked to select predefined bins (Under 21, 21–34, 35–44, 45–54, 55–64, and 65 years and over), and for fishing experience, were asked to do the same (5 years, 10 years, 15 years, 20 years, 30 years, and 30 + years).

The core of the survey was an exercise where respondents were asked to evaluate or score the quality of bonefishing for the area they fished currently or had fished previously. This exercise employed a life history calendar approach by providing survey respondents with a matrix of temporal events across spatial domains (see Rehage, et al.^42^ for further details). For time, respondents were asked to evaluate the quality of bonefishing at present, and 5, 10, 15, 20, 30 and 40 years ago. These increments corresponded to seven focal time steps that were used for analysis (1975, 1985, 1995, 2009, 2005, 2010, and 2015). For the spatial domain, five focal regions were included (Biscayne Bay, Florida Bay, Uppers Keys, Middle Keys, and Lower Keys). Due to the design of the survey, respondents were only allowed to evaluate bonefishing over their own personal fishing history and spatial domain. For example, a recreational angler that fished for the past 20 years (2015–1995) in Florida Bay and the Upper Keys was only given the opportunity to evaluate the quality of bonefishing across the five time steps encompassing their fishing history and two regions, totalling 10 entries in the matrix.

Using this format, respondents were asked to score the quality of bonefishing in terms of three metrics: (1) the overall bonefish fishing quality (number of fish seen, opportunities to cast, fish caught, size of schools and size of fish (2) bonefish fishing quality in terms of the size of bonefish, and (3) bonefish fishing quality in terms of the number of bonefish they had the opportunity to catch. Bonefishing is a sight fishery where anglers cast only after encountering fish, such that fishing quality scored by a fisher or guide is a combination of fish observed, encounters by the fishing vessel, attempts to catch, as well as actual catch (all best captured by shots or opportunities to cast). Each matrix used a five-point Likert scale (one for the lowest quality and five for the highest quality). The current analysis used only the question on overall bonefish fishing quality. Analysis of bonefish fishing quality in the additional metrics of size and opportunities to catch (shots) are presented in Rehage, et al.^42^.

### Data analysis

Our analysis comprised three parts: (1) aggregation of fishing quality estimates and comparisons of estimates among homogenous groups, (2) analysis to ascertain sample size thresholds using saturation methods, and (3) comparison of fishing quality estimates among smaller groups with varying diversity distributions.

For the first part of our analysis, and in the absence of any formal data on stocks and trends for bonefish in South Florida, we treated results from the full sample of the survey described above (n = 210 respondents) as the *true* trend to compare against. We assume these results to be true, but acknowledge limitations given the size of the fishery, which includes roughly 300 fishing guides^47^. Importantly though, the trend matches other forms of data for the region, including tournament landings^42–47^, but no other fisheries-dependent or -independent data exists for the time period in question. Given that answers to the question on bonefish fishing quality were on a five-point Likert scale (one for the lowest quality and five for the highest quality), our aggregation process involved averaging responses over the 7 time periods described above to produce mean quantitative estimates of bonefish fishing quality^26^, ranging from 1 to 5 (Supplementary Fig. 2). This approach counters calculating proportions of responses to each score and visually presenting it, as one would normally do for Likert-type data (Supplementary Fig. 5). Then, in order to examine differences among homogenous groups based on three traits, age, fishing experience, and resource use (i.e., recreation or livelihood), we aggregated responses in the same way, but for each individual group. For example, we grouped respondents by age class, and produced separate response averages over the 7 periods.

Combined with visually comparing differences in the trends produced by each homogenous group (e.g., based on three traits vs. the full sample), we used relative absolute error metrics that are frequently used to evaluate the accuracy of time series predictions^70^, and of fishery stock models^71^. The accuracy of the bonefish fishing quality estimates from homogenous groups were assessed based on the mean absolute percentage error (MAPE) between each of the three groups (based on age, experience and guide vs. angler) and the full sample. We expressed this as a percentage value defined by the formula:

$$MAPE = \frac{{\sum \frac{{\hat{y}_{i} - y_{i} }}{{y_{i} }}}}{n}$$

where $\hat{y}_{i}$ is the individual estimate for the i^th^ time point by a respondent group and y_i_ is full sample estimate for the i^th^ time point. Their difference is divided by the full sample estimate y_i_. The absolute value of this ratio is summed for every individual in time and divided by the number of scores *n*. Values < 5% are typically considered highly accurate (i.e., the sub-sample estimate is close to the full sample estimate). This outcome would mean that the estimate of bonefish fishing quality derived from a smaller group of homogenous respondents was similar to that derived from the full sample of diverse respondents (n = 210). For the purpose of this study, we considered values of the MAPE between 5 and 10% are good and values between 10 and 20% reasonably accurate., while values > 20% are considered poor, suggesting that the estimate from the sub-sample differs strongly from the overall or ‘true’ estimate.

In the second part of our analysis and to ascertain sample size thresholds for bonefish fishing quality estimates, we combined rarefaction methods used in community ecology^72,73^ with saturation principles applied in social science analyses^68,74^. Rarefaction methods are well-developed approaches to control for sample size effects on biodiversity estimates^67^. Correspondingly, thematic saturation principles account for sample size effects on accumulation of new ideas, information or themes encountered across a series of individuals in a social science survey^75^. In a broad sense, the approaches have very similar goals; to ascertain whether enough samples were collected to ensure that all species (in ecology) or ideals (in social science) were recorded. Most strategies used to assess saturation in social science depend on simple counts of the number of themes presented, and rarely use approaches to randomly iterate samples as used for rarefaction in ecology^74^. To operationalise our approach, we first used repeated random subsampling^76^ to evaluate how the number of respondents influenced the maximum number of unique responses to the question of bonefish fishing quality. Repeated random subsampling resamples a single data set *without* replacement (thus maintaining unique responses) to create many randomly selected subsets, which then are compiled over multiple (random) iterations^77^. Because an individual can only be surveyed once, this saturation method differs from bootstrap resampling (where the resampling is *with* replacement).

To produce saturation curves, each iteration used a random subset of respondents and their answers to the question of bonefish fishing quality. For each random iteration, we calculated the number of unique responses for each time period. Sample size ranged from one to the maximum number of respondents (210), and we performed 10,000 random iterations. For each year, we graphed sample size against the number of unique responses based on this repeated random subsampling to create unique response saturation curves and used these curves to identify periods in the time series with large enough sample sizes to reach saturation in responses. Based on the maximum number of possible responses, we then calculated the mean sample size (across all time periods) needed to capture 100%, 75% and 50% of unique responses.

For the final part of our analysis, aiming to compare bonefish fishing quality estimates among smaller groups with varying diversity distributions, we used mean sample sizes needed for 75% and 50% of unique responses for a second phase of repeated subsampling. Rather than random subsampling, this second process used repeated non-random subsampling. We produced a range of sampling distributions based on two of the respondent traits: age structure and fishing experience (Supplementary Fig. 6). For age structure, we produced three sample (or crowd) distributions where (a) age was uniform (equal proportions for each age class), (b) age was skewed towards younger individuals (younger crowd), and (c) where age was skewed towards older individuals (older crowd). For fishing experience, we also produced three crowd distributions where fishing experience was uniform (equally experienced), experience was lower (less experienced), and experience was greater (more experienced). For each iteration of the subsampling process, rather than randomly choosing individuals, the likelihood of picking an individual was based on the crowd distribution. For example, for the older crowd, the likelihood of sampling individuals increased with their age, and for the less experienced crowd, the likelihood of sampling individuals decreased with their experience. We performed 200 iterations for each crowd distribution, as well as 200 iterations for a random distribution; 100 at the sample size needed to capture 75% of unique responses, and 100 at the sample size needed to capture 50% of responses. As with the homogenous groups, the accuracy of the bonefishing quality estimates from each crowd distribution subset were assessed based on the MAPE, again compared with the full sample. All statistical analysis and graphing were conducted in R^78^.

## Supplementary Information

Supplementary Information.

## Data Availability

The datasets and code used for analysis is available on figshare: https://doi.org/10.6084/m9.figshare.28060571. All identifiable information has been removed from the dataset.
