# Supplementary material for: Stakeholder diversity matters: employing the wisdom of crowds for data-poor fisheries assessments
Source: Sci Rep. 2025 Jan 2;15:440. doi: 10.1038/s41598-024-84970-4 (PMC11696029; doi:10.1038/s41598-024-84970-4)
Supplement: Supplementary file 1 — Supplementary Information. [file 41598_2024_84970_MOESM1_ESM.docx]

**Supplementary material**


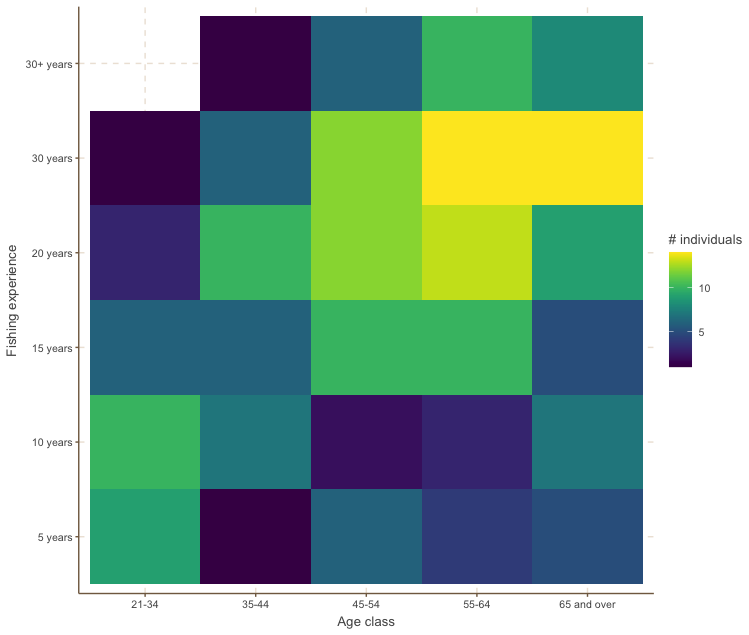


**Supplementary Fig. 1.** Tile plot showing distribution of respondents across fishing experience categories and age classes. If age and fishing experience were related, we would expect to see a diagonal yellow band in the middle, radiating to purple in the top left and bottom right of the plot.


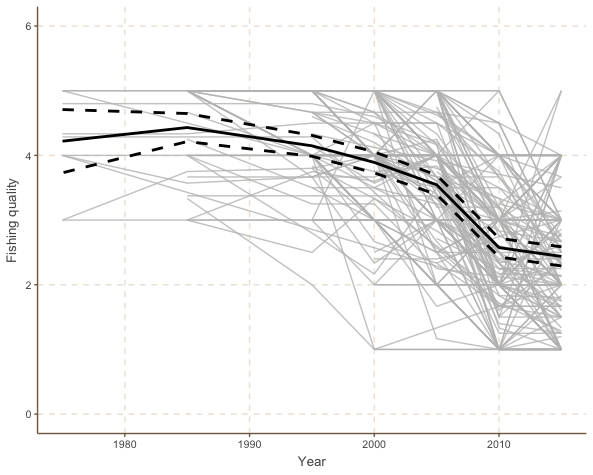


**Supplementary Fig. 2.** Aggregated estimates of bonefish fishing quality in South Florida, USA, from 1975-2015, where a value of 5 = very good and a value of 1 = very poor. The black line represent the mean of all 210 respondents (± 95% confidence intervals represented by black dashed lines) and grey lines show all 210 individual estimates.


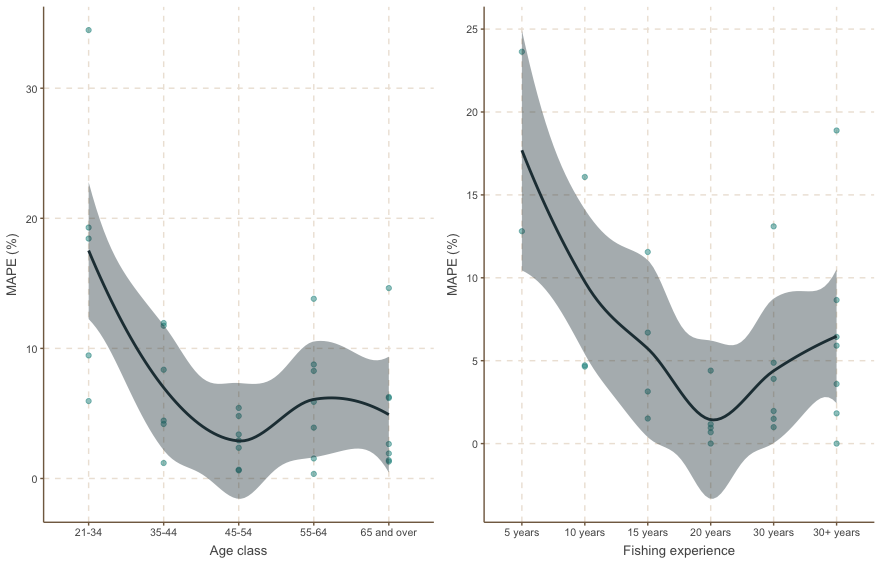


**Supplementary Fig. 3.** Scatter plots showing non-linear relationships between mean absolute percentage error (MAPE) and age class (left) and fishing experience (right).


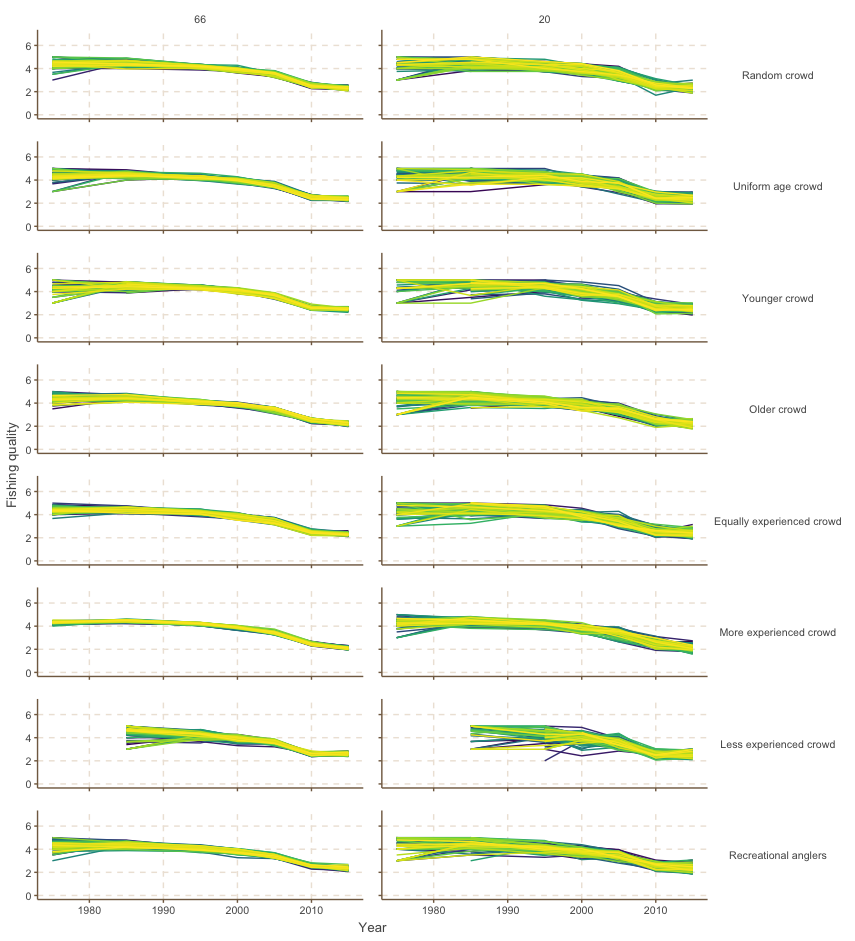


**Supplementary Fig. 4.** Aggregated estimates of bonefish fishing quality in South Florida, USA, from 1975-2015 produced by 100 random subsets of the number of individuals needed to produce 75% of unique responses (n=66) and 50% of unique responses (n = 20). Each line represents a different subset, and each paired panel represents a different crowd distribution.

**
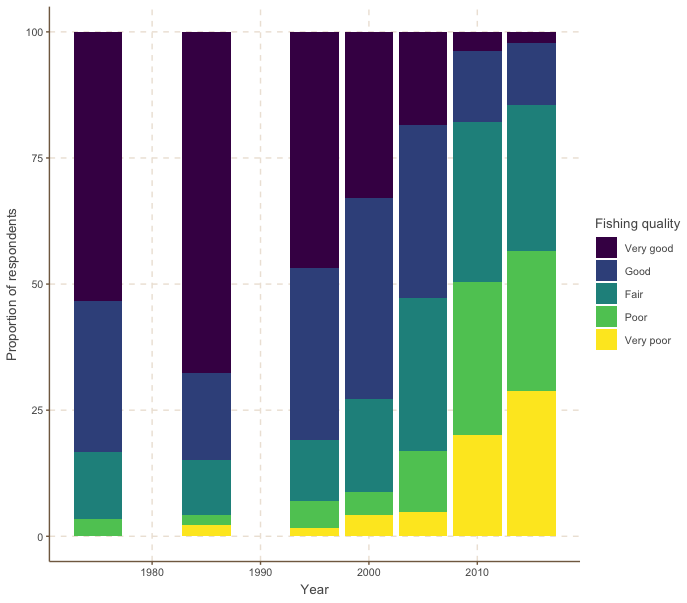
**

**Supplementary Fig. 5.** Proportions of responses to each fishing quality score across periods.


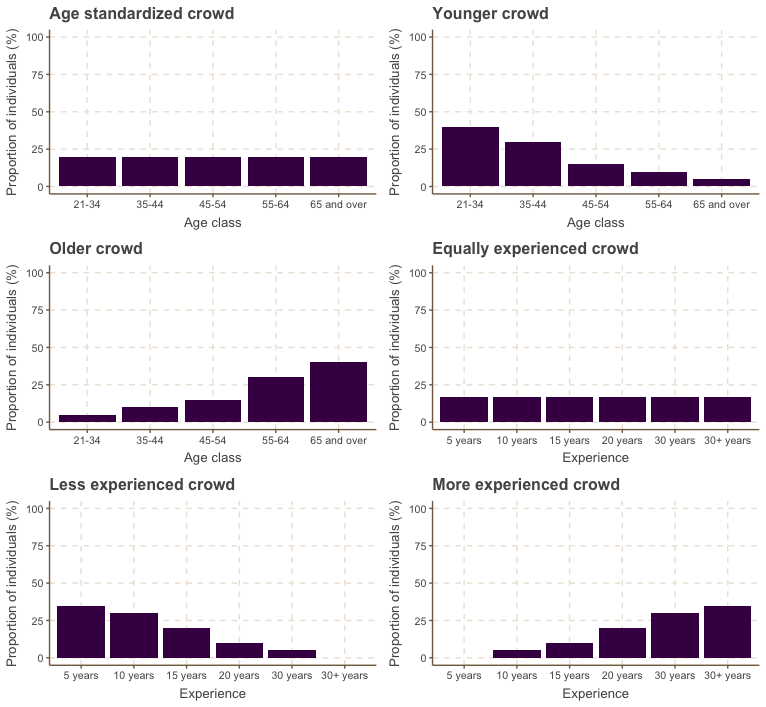


**Supplementary Fig. 6.** Crowd sampling distributions used for repeated non-random subsampling.
